# Combined Aerobic and Strength Training Improves Dynamic Stability and can Prevent against Static Stability Decline in Postmenopausal Women: A Randomized Clinical Trial

**DOI:** 10.1055/s-0043-1772178

**Published:** 2023-09-08

**Authors:** Ana Claudia Fortaleza Marques, Fabrício Eduardo Rossi, Lucas Melo Neves, Tiego Aparecido Diniz, Iracimara de Anchieta Messias, José A. Barela, Fay B. Horak, Ismael Forte Freitas Júnior

**Affiliations:** 1 Department of Medicine, Universidade do Oeste Paulista, Presidente Prudente, SP, Brazil; 2 Immunometabolism of Skeletal Muscle and Exercise Research Group, Department of Physical Education, Universidade Federal do Piauí, Teresina, PI, Brazil; 3 Universidade Santo Amaro, São Paulo, SP, Brazil; 4 Department of Psychiatry, Faculdade de Medicina da Universidade de São Paulo, São Paulo, SP, Brazil; 5 Universidade de São Paulo, São Paulo, SP Brazil; 6 Faculty of Science and Technology, Universidade Estadual Paulista “Júlio de Mesquita Filho,” Presidente Prudente, SP, Brazil; 7 Department of Physical Education, Instituto de Biociências, Rio Claro, SP, Brazil; 8 Department of Neurology, Oregon Health & Science University, Portland, OR, United States

**Keywords:** physical exercise, menopause, balance, concurrent training, gait, exercício físico, menopausa, equilíbrio, treinamento concorrente, marcha

## Abstract

**Objective** To analyze the effect of combined training (CT) in postural control and gait parameters in postmenopausal women.

**Methods** A parallel-group, randomized, control study was conducted with 16 weeks of combined training ( *n* = 16) versus a non-training control group ( *n* = 12) in postmenopausal women (aged 59.3 ± 8.0). Pre and postintervention assessments included postural control (using an AMTI force platform – Advanced Mechanical Technology, Inc., Watertown, MA, USA) and gait impairments (using baropodometry). In addition, the upper limb strength and abdominal tests, as well as aerobic capacity, assessed functional indicators.

**Results** The CT intervention in postmenopausal women resulted in improved gait (stride length ( *p* = 0.006); speed ( *p* = 0.013); double support time ( *p* = 0.045); and improved postural control (displacement area of postural sway in a normal base of support with eyes open ( *p* = 0.006). Combined training increased functional indicators (abdominal - *p* = 0.031; aerobic capacity - *p* = 0.002).

**Conclusion** In conclusion, combined aerobic plus strength training effectively improved gait and balance control in older women. The postmenopausal women from the CT group walked faster and with bigger steps after the intervention than the control group. In addition, they presented decreased postural sway in standing and decreased the percentage of double support time while walking, which means improved static and dynamic balance control and functional indicators.

## Resumo

**Objetivo** Analisar o efeito do treinamento combinado (TC) no controle postural e nos parâmetros da marcha em mulheres na pós-menopausa.

**Métodos** Foi realizado um estudo controlado randomizado de grupos paralelos com 16 semanas de treinamento combinado ( *n* = 16) versus um grupo controle sem treinamento ( *n* = 12) em mulheres na pós-menopausa (59,3 ± 8,0 anos). As avaliações pré e pós-intervenção incluíram controle postural (usando a plataforma de força AMTI) e deficiências da marcha (usando baropodometria). Além disso, os testes de força de membros superiors e abdominal, bem como a capacidade aeróbica, avaliaram indicadores funcionais.

**Resultados** A intervenção do TC em mulheres na pós-menopausa resultou em melhora da marcha (comprimento da passada ( *p* = 0,006), velocidade ( *p* = 0,013), tempo de apoio duplo ( *p* = 0,045) e controle postural aprimorado (área de deslocamento da oscilação postural em base de apoio normal com olhos abertos ( *p* = 0,006). O TC aumentou os indicadores funcionais (abdominal - *p* = 0,031; capacidade aeróbia - *p* = 0,002).

**Conclusão** Em conclusão, o TC de força e aeróbico melhorou efetivamente o controle da marcha e do equilíbrio em mulheres idosas. As mulheres na pós-menopausa do grupo CT caminharam mais rápido e com passos maiores após a intervenção do que o grupo controle. Além disso, elas apresentaram redução da oscilação postural em pé e do percentual de tempo de apoio duplo durante a caminhada, o que significa melhora no controle do equilíbrio estático e dinâmico e dos indicadores funcionais.

## Introduction

Postmenopausal women present postural control impairments when standing across a variety of conditions (e.g., bipedal and semi-tandem; eyes open and closed). 1 2 3 4 5 6 7 8 9 10 Gait impairments are also observed in this population, with slow gait velocity and long double support time. 11 12 Impairments in postural control and gait may result in difficulty in managing daily activities and increase the risk of falls. Sociodemographic (e.g., age) and functional changes (e.g., previous falls) are associated with these impairments in postural control and gait. 13 In addition, postmenopausal women's postural control and gait performance can be influenced by other health and physical indicators such as body mass index, body composition changes, and physical fitness. 14 Many of these physical indicators can be avoided and/or minimized by physical exercise programs, an important non-pharmacological strategy. 15

Different types of physical exercise programs have been shown to improve the health and physical indicators in postmenopausal women, 16 17 18 19 20 21 22 23 24 25 and promote improvements in postural control 22 23 and gait performance. 24 25 Specifically, 8 weeks of aerobic exercise intervention effectively improved several aspects of balance control in older women. 23 In addition to the benefits of aerobic exercise, a meta-analysis indicated that strength exercises promote significant and large effect size (0.84–confidence interval = 0.52–1.56) improvements in gait velocity. 25 In addition, improvements in leg strength after strength exercise were superior to those observed after aerobic exercise. After strengthening exercises, leg extension and flexion strength improvements may contribute to meaningful improvements in static and dynamic balance control. 26

The impact of different exercise programs in postural control and gait in older women is promising. Moreover, there is a need to further understand and examine the possible positive effects of other exercises. For Instance, we have shown that combining aerobic and strength exercises (combined training - CT) is important to improve postural control after 12 weeks of training in older women (over 60 years old). 27 Based upon all these results, we wondered about the possible benefits of CT on posture and gait for postmenopausal women.

Due to metabolic changes, postmenopausal women experience an accumulation of total, hip, and trunk fat, which affects posture, 28 leading to an increase in the risk of falls and fractures, 3 and this condition may be associated with frailty. 29 The CT is an efficient training strategy for changing body composition, increasing lean mass, and reducing fat, especially in the trunk. 19 Therefore, CT might constitute an important training protocol to improve postural control and gait parameters. Thus, the purpose of this study was to examine the effects of a 16-week CT protocol on postural control and gait performance in postmenopausal women.

## Methods

This study was a prospective, parallel-group, randomized, controlled study with 16 weeks of CT versus a control (C) group. Subjects had pre and postintervention anthropometric, postural control and gait performance assessments, and functional indicators performed during the week before and after the interventions. All the procedures were approved by the Institutional Ethical Committee (protocol 388.070), following the Declaration of Helsinki. 30 Also, the study was registered in the Brazilian databases of clinical trial (RBR-9CBP8S). Furthermore, all participants agreed and signed the consent form prior to enrolment in the study.

Subjects were invited through newspaper and television advertising to participate in the study, and, after phone contact, an appointment was scheduled for a more detailed screening interview. The inclusion criteria were: (a) postmenopausal women, without a menstrual cycle for at least 1 year; (b) to be between the ages of 50 and 79 on the date of the evaluation; (c) medical authorization to participate in the training; (d) no physical limitations or health problems that could prohibit the completion of the assessments and exercise intervention (e.g., uncontrolled diabetes, hypertension, or rheumatoid arthritis); (e) no participation in any systematic physical exercise for at least 6 months before the study; (f) no history of hormone replacement therapy. The exclusion criteria were accumulated 3 consecutive absences or 4 non-consecutive absences in the intervention for 1 month. After this initial screening, participants were allocated randomly to one group (CT or C). 31 Simple randomization techniques were used for allocation (1:1), which ensures that trial participants have an equal chance of being allocated to a treatment group by a researcher who was blinded to the group allocation.

**Fig. 1:**
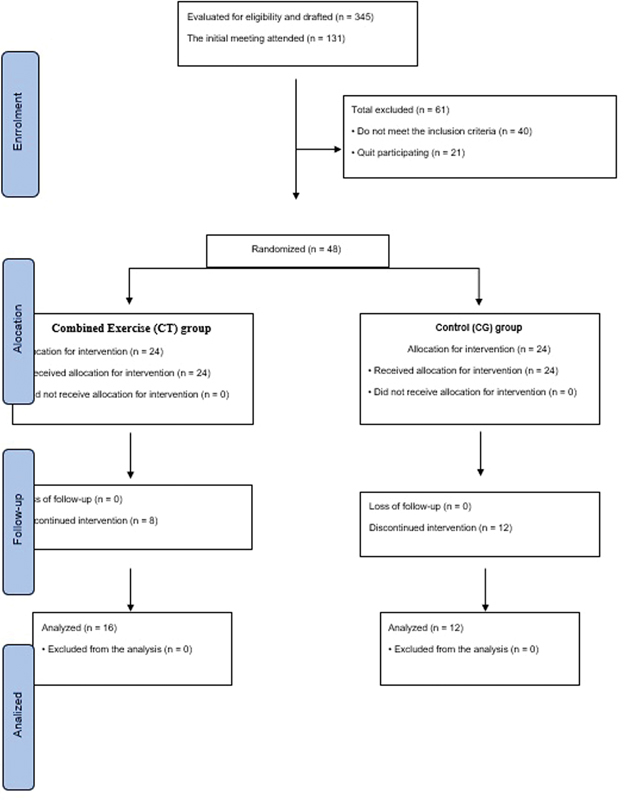
Consolidated Standards of Reporting Trials (CONSORT) flowchart of study participants through the 16-week study. CT, combined training; CG, control group.

Fig. 1 (Consolidated Standards of Reporting Trials [CONSORT] flowchart) shows the recruitment procedures. A total of 361 postmenopausal women registered for the first call, and 131 attended the initial screening meeting. Of these, 61 postmenopausal women were excluded (40 postmenopausal women did not meet the inclusion criteria and 21 refused to participate in the study). After this initial screening, 48 postmenopausal women performed baseline tests and were randomized to one of the groups (CT or C). The participants allocated to the CT group should present medical authorization to participate in the training routine. During the intervention period, 20 participants dropped out of CT ( *n* = 8), dropped out of 33.3% and C ( *n* = 12), dropped out of 50%. The reasons for dropouts included health problems, personal/family problems; unspecified reasons, which led to failure to participate in the final assessment. Using an effect size for gait velocity (partial eta squared = 0.24), an α value of 5%, a 98% power to our sample size was detected.

The CT group (age = 59.1 ± 8.1 years; height = 155.8 ± 6.4 cm) involved strength and aerobic training in each exercise session. Subjects exercised for 90 minutes, 3 times per week for 16 weeks. Before each exercise session, participants performed 5 minutes of warm-up exercises and 5 minutes of stretching at the end of the training session.

Before starting the program, participants performed 2 weeks of equipment and training routine familiarization (without load). The exercises were performed 3 times a week (Monday, Wednesday, and Friday) for 90 minutes a day. They were comprised of 5 minutes of warm-up, 50 minutes of resistance training, 30 minutes of aerobic training, and 5 minutes of stretching at the end. After that, participants exercised for 16 weeks, keeping the same frequency and time. All sessions were supervised by a professional of physical education or physiotherapist. The protocol training is described below.

Strength training was composed of the following exercises: leg extension; 45° leg press; leg curl; bench press; seated row; triceps extension; arm curl; arm side elevation with dumbbells, and abdominal exercises, according to Rossi et al. 19 The exercise routine was performed always in the same gym. The intensity of strength training was controlled through the zone of maximum repetitions. The series was executed until momentary exhaustion (e.g., the repetitions should be between 12 to 15 repetitions maximum). The load was increased when the participants executed more than 15 repetitions to have the training zone respected. 19 The strength training program consisted of 4 progressive phases: phase 1–1 ^st^ to 4 ^th^ weeks, 12 to 15 repetitions, 3 sets per exercise; phase 2: 5 ^th^ to 8 ^th^ weeks, 10 to 12 repetitions, 3 sets per exercise; phase 3–9 ^th^ to 12 ^th^ weeks, 8 to 10 repetitions, 3 sets per exercise; phase 4–13 ^th^ to 16 ^th^ weeks, 8 repetitions, 3 sets per exercise. For all weeks, the participants had a 60-second break between sets. The 20-point scale, as standardized by Borg et al., 32 was used to determine the rate perceived effort (RPE) after each training session.

Aerobic training involved walking in an external environment. The intensity was performed using the critical velocity protocol, 33 34 already used in our previous studies with postmenopausal women. 16 20 35 36 The critical velocity was determined by having subjects walk as quickly as possible 3 different distances (400, 800, and 1,200 m) on a running track on 3 different and non-consecutive days. The time for performing the distance was recorded (digital stopwatch – model S810i or RS800, Polar Electro, Espoo, Finland). The relationship between the distance (m) and the exercise time (s) was linearly adjusted, and we assumed the critical velocity to be the slope of this model, 37 which is the intensity of aerobic training. 38 Women walked at 60 to 70% from their critical velocity during the training sections.

The participants were instructed to refrain from structured train–ng program and to maintain their regular dietary intake during the intervention.

The C group (age = 59.7 ± 8.2 years; height = 155.8 ± 6.7 cm) were instructed to avoid changing their activities or starting any new exercise program for 18 weeks (between the initial and final assessments of the research).

Anthropometric measurements were composed of body weight and height measurements. Bodyweight was obtained using an electronic scale (Filizola PL 50 - Filizola Ltda., Fortaleza, CE, Brazil) (accuracy of 0.1 kg) and height using a stadiometer (Sanny, São Paulo, SP, Brazil) (accuracy of 0.1 cm and length of 2.20 m).

Postural control during standing was tested by measuring postural sway using an AMTI force platform (model-BP600400–Advanced Mechanical Technology, Inc., Watertown, MA, USA) and AMTI-NetForce software (Advanced Mechanical Technology, Inc.. The participants were evaluated under two support bases (normal base with feet parallel at shoulder width and semi-tandem stance) and two visual conditions (eyes open and eyes closed), resulting in four conditions.

The participants were asked to stand upright on the force platform, barefoot, and stand as still as possible, with arms down at the sides of the body for 30 seconds. In the eyes-open condition, they were asked to fixate on a target (white tape – 2 × 5 cm) placed on the wall, 2-m away, at their eye level. In the eyes-closed condition, participants keep their eyes open in the dark while wearing a pair of goggles covered with black tape, preventing the availability of any visual cues. The order of the conditions was randomly defined.

The center of pressure displacement was recorded at a frequency of 100 Hz. Customized routines written in MATLAB (The MathWorks Inc., Natick, USA) were used to filter (second-order Butterworth digital filter, cut off frequency of 5 Hz) the center of pressure data in both medial-lateral (ML) and anterior-posterior (AP) directions. The center of pressure ellipse area (95% of total area) was used to quantify postural control.

The gait performance was assessed using a 2-m baropodometry gait mat (FootWork Pro - AM cube, Gargas, France). Participants walked along an 8-m walkway, in which the baropodometry mat was arranged in the center with 3m before and after it for acceleration and deceleration, respectively. The participants were instructed to walk at their preferred velocity throughout the walkway, performing three repetitions each way. Data from the baropodometry were obtained at 200 Hz frequency and analyzed by Software Footwork Pro (IST Informatique, Gargas, France). Gait performance was quantified as stride length, stride time, gait velocity, and double support time (percent of gait cycle). 39

As functional indicators, we adopted the tests of a) upper limb strength, b) Abdominal, and c) Aerobic capacity. This protocol was described in a preview study. 40

Normality assumption was confirmed (Shapiro-Wilk test), the estimated sphericity was veriﬁed (Mauchly's W test), and, when necessary, the Greenhouse-Geisser correction was used. For each outcome measure, a mixed between-group–within-subject multivariate analyses of variance (MANOVA and ANOVA) were employed, having as factors, group (control and CT) and the two evaluation sessions (pre and posttraining), this last factor treated as a repeated measure. When necessary, univariate analyses and posthoc tests, with Bonferroni adjustments, were employed. The partial eta squared (η ^p2^ ) was reported for time, group and interaction effects, and the threshold values were > 0.001 (small), > 0.06 (moderate), and > 0.14 (large). All statistical analysis was performed using the IBM SPSS Statistics for Windows, version 21.0 (IBM Corp., Armonk, NY, USA). We adopted a significance level *p* ≤ 0.05.

## Results

Table 1 presents mean (± SD) values of body weight and functional indicators for both groups and evaluations and the group. There were no statistically significant differences between groups at baseline for all variables investigated ( *p* > 0.05). For functional indicators, there were statistically significant interactions in the abdominal test ( *F* = 5.53, *p* = 0.029, η ^p2^ = 0.22) and aerobic capacity ( *F* = 6.78, *p* = 0.017, η ^p2^ = 0.25). Posthoc tests showed that the CT group increased repetitions in the abdominal test posttraining compared with baseline ( *p* = 0.031) with higher values than the C group ( *p* = 0.006), and the CT group showed lower time in seconds in the aerobic capacity compared with the C group ( *p* = 0.002). For muscle strength in the upper limb, there was a main effect of time ( *F* = 17.23, *p* < 0.001, η ^p2^ = 0.46) and significant difference between groups ( *F* = 8.20, *p* = 0.010, η ^p2^ = 0.29) but no interaction was observed. There were no main effects of time or statistically significant differences between groups or interactions for body weight ( *p* > 0.05).

**Table 1:** Mean (± SD) values of body weight and functional indicators for both groups and evaluations and the group and evaluation interaction *p* -values

Variables	Control group ( *n* = 12)		CT group ( *n* = 16)		*P* -value
	Pre	Post	Pre	Post	
Body weight (kg)	65.0 ± 11.4	65.5 ± 11.6	68.1 ± 8.9	68.1 ± 9.1	0.446
Muscle strength (repetitions)	20.2 ± 4.3	22.6 ± 4.0	23.5 ± 3.5	27.1 ± 2.8	0.332
Abdominal (repetitions)	6.0 ± 8.7	4.7 ± 5.7	6.1 ± 9.5	13.1 ± 9.3 ^*£^	0.029
Aerobic capacity (seconds)	499.2 ± 44.6	503.8 ± 41.1	500.4 ± 27.5	469.4 ± 40.3 ^£^	0.017

Abbreviations: CT, combined training.

*= Bonferroni posthoc test with *p* -value < 0.05 compared with Pre; £= Bonferroni posthoc test with *p* -value < 0.05 compared with the control group.

**Fig. 2:**
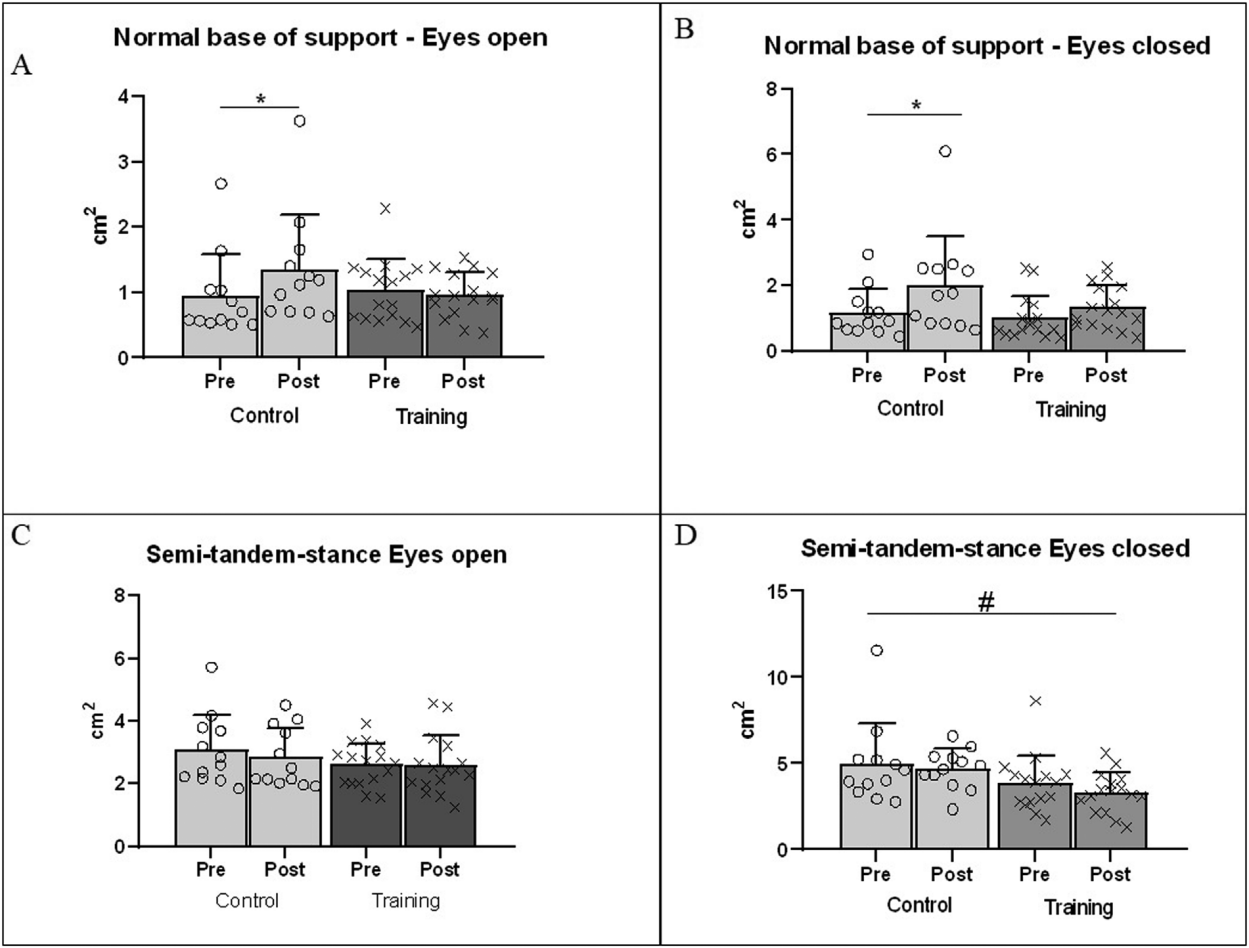
Comparation of center of pressure ellipse area. CT, combined training – Panel A: Normal base of support eyes open. Panel B: Normal base of support eyes closed. * = Bonferroni posthoc test with *p* -value < 0.05 compared with pre. # = main difference between groups.

Fig. 2 depicts postural sway ellipse area mean values for both groups and evaluations for both stance position and visual conditions. In the normal stance position, MANOVA revealed significant pre-post assessment effect (Wilks Lambda = 0.697, F (2.25) = 8.10, *p* = 0.002) with a significant group and assessment interaction (Wilks Lambda = 0.736, (2.25) = 4.48, *p* = 0.02). Univariate analyses showed an effect of assessment occurred for both eyes open (panel A) ( *F* = 5.05, *p* = 0.033, η ^p2^ = 0.16) and eyes closed (panel B) ( *F* = 16.39, *p <* 0.001, η ^p2^ = 0.38) conditions showed that the ellipse area was larger in the post than in the preassessment. Additionally, univariate analyses showed that an interaction effect occurred only in the eyes-open condition ( *F* = 8.967, *p* = 0.006, η ^p2^ = 0.25), and Bonferroni posthoc tests showed a significant ellipse area increase in the C group, postevaluation ( *p* = 0.002), but no difference in the CT group. In the semi-tandem position with eyes open (panel C), there was no significant difference (Wilks Lambda = 0.786, F (2.25) = 3.39, *p* = 0.543), but univariate analyses showed an effect of group only in the eyes-closed condition ( *F* = 5.356, *p* = 0.029, η ^p2^ = 0.17), with the C group showing larger sway than the CT group (panel D).

**Fig. 3:**
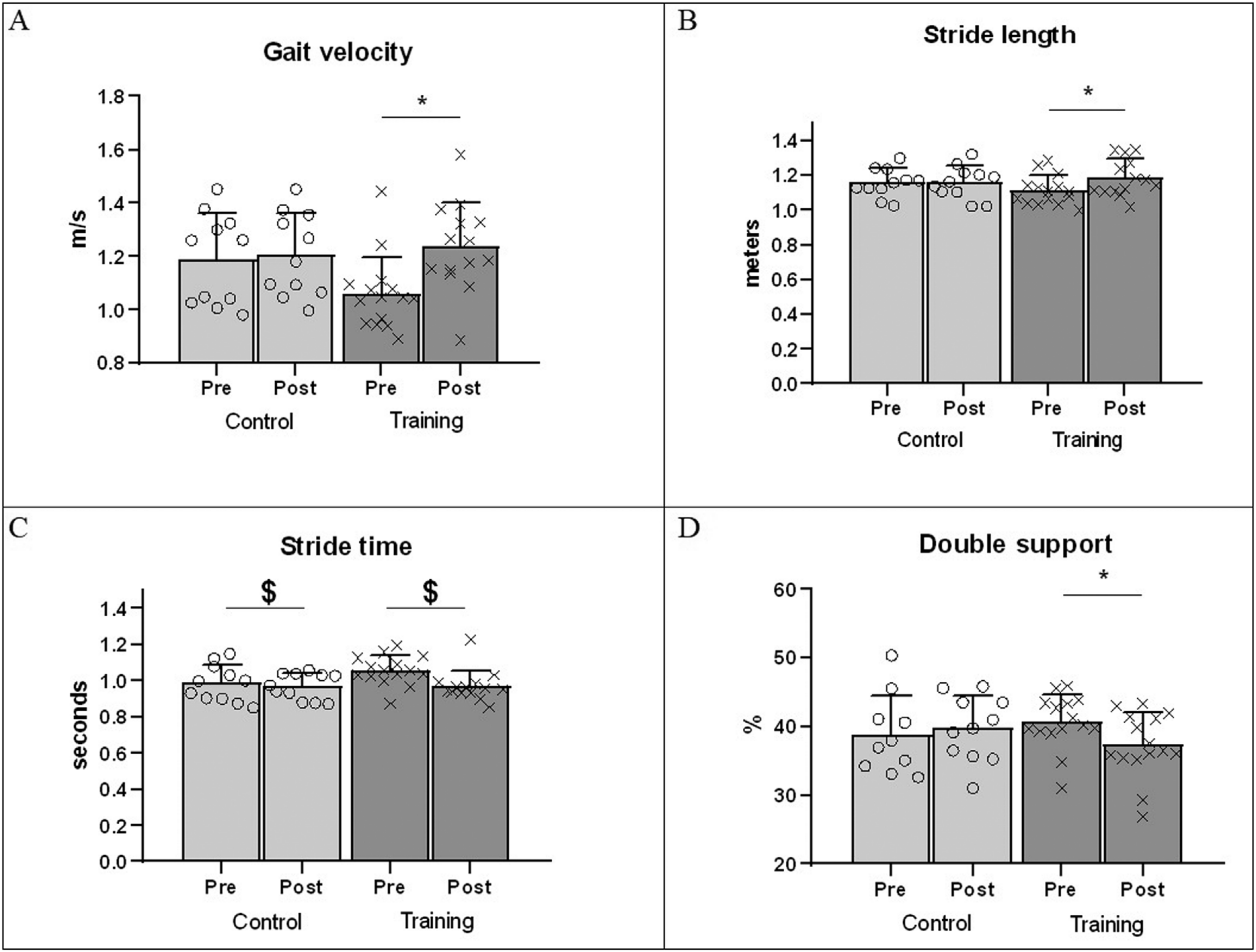
Comparation of gait parameters. m/s = meters per second. CT = combined training – Panel A: Gait velocity; Panel B: Stride length; Panel C: Stride time; Panel D: Double support. * = Bonferroni posthoc test with *p* -value < 0.05 compared with pre. $ = Main effect of assessment.

Fig. 3 depicts values of gait performance for both groups, pre and postevaluation. For gait velocity (panel A), ANOVA showed a significant pre-post assessment effect ( *F* = 10.432, *p* = 0.004, η ^p2^ = 0.31) and a significant group and assessment interaction ( *F* = 7.169, *p* = 0.013, η ^p2^ = 0.24). Posthoc tests showed that the CT group increased gait velocity postevaluation ( *p* < 0.001). Similar results were observed for the stride length (panel B), with ANOVA revealing evaluation effect ( *F* = 10.173, *p* = 0.004, η ^p2^ = 0.31), and group and assessment interaction ( *F* = 9.037, *p* = 0.006, η ^p2^ = 0.28). Posthoc tests showed that the CT group increased stride length postassessment ( *p* < 0.001 *)* . For stride time (panel C), ANOVA revealed only a significant assessment effect ( *F* = 7.294, *p* = 0.013, η ^p2^ = 0.24), with both groups reducing stride duration, when comparing post and preevaluation. Finally, for the double support time (panel D), ANOVA revealed only a group by evaluation interaction ( *F* = 4.508, *p* = 0.045, η ^p2^ = 0.16). Posthoc tests showed that the CT group reduced double support duration post-evaluation ( *p* = 0.006).

## Discussion

This study aimed to examine the effects of a 16-week of CT training protocol on postural control and gait performance in postmenopausal women. The 16 weeks of CT intervention improved gait performance in this cohort. In addition, postural sway increased in the C group but not the CT group. To the best of our knowledge, this is the first study to show the effects of CT on gait and postural control in postmenopausal women.

Postmenopausal women walked faster, with longer steps and with shorter double support time after the CT intervention. Conversely, post-menopausal women from the control group did not show any gait change. Dias et al. also observed an increase in gait speed in postmenopausal women after 12 weeks of 2 strength-training protocols (cluster-set and traditional inter-repetitions rest method). 41 Our results, however, add to the existing knowledge showing that such walking velocity increase was due to longer steps and a tendency of shorter stride duration. Thus, the CT intervention promotes several improvements in gait parameters that allow postmenopausal women to walk faster.

Our results also showed that postmenopausal women decreased the double support duration after the CT training protocol compared with pretraining. A shorter double support duration during walking improves gait stability. Aragão-Santos et al. also found gait speed increase in postmenopausal women after element-based functional and task-specific-based functional training protocols but did not find improvements in gait stability. 42 Thus, the CT training seems to contribute even further than other interventional protocols in terms of improving gait stability, but this issue requires future research. Another important issue related to the CT protocol employed in this study was that postmenopausal women performed the aerobic training walking in an external environment, which improved aerobic capacity by providing a specific stimulus for neural adaptations required for walking in the real world. 26

The CT protocol did not impact the postural sway, although the C group increased their postural sway after 12 weeks without intervention. The center of pressure displacement ellipse area, under the normal base of support and eyes-open condition, showed that while women enrolled in the 16-week CT maintained the same ellipse area, women in the C group increased the ellipse area. Despite being characterized by large variability, a similar tendency ( *p* = 0.090) was observed in the normal base with eyes closed. Thus, the CT employed in this study avoided performance deterioration in postmenopausal women when standing in a normal position. Such observation corroborates previous suggestions that exercise prevents postmenopausal 'women's postural control system deterioration, 43 and, especially in women over 50 years. 44

We hypothesized that CT could improve muscle strength leading to better postural control performance. 45 However, our results did not improve postural sway between pre and posttraining in the normal stance and semi-tandem stance, although CT improved functional indicators observed by the number of repetitions in the abdominal test and aerobic capacity; thus, the increase in strength in the abdominal region could have influenced postural control performance in the CT group, since, in this region, both the external and internal forces act directly to accelerate or decelerate the body. Perhaps a more challenging postural condition, such as tandem or one-legged stance, would have improved postural control with increased leg and trunk strength. Nevertheless, the CT group showed better postural control performance than the C group, which corroborates results observed after 32 weeks of strength training. 26 Furthermore, besides the improvements in gait and lack of deterioration of postural control showed in the present study, women who practice CT have other benefits demonstrated in other studies, such as improvements in body composition, with a decrease in body fat mass and increase in lean mass, 19 and improvements in strength 46 and cardiovascular condition. 47

Our study has limitations: the absence of lower limb muscle strength and body composition assessments. In addition, the functional test used in the present study can be enough toidentify an improvement in upper body strength; therefore, maybe a more specific test, such as the 1RM test, would have shown better benefits for upper body strength. Nevertheless, the present study contributes to the literature, since professionals who work with postmenopausal women should include combined aerobic and strength exercises that may improve gait speed and stability. Improvement in dynamic stability when walking can reduce the risk of falls, fractures, 48 consequent hospitalization as well as decrease difficulty in mobility and in the performance of daily activities. 49 Furthermore, we suggest that future randomized control trials are performed, analyzing gait and balance control according to body composition and fitness capacity.

## Conclusion

Combined training (aerobic plus strength) improved gait variables and avoided the postural control decline after 16 weeks of intervention in postmenopausal women. In addition, the women from the CT group walked faster and with bigger steps after the intervention than the those in the C group, and they decreased the percentage of double support time and showed improvement of functional indicators.
